# IGFBP7 and GDF-15, but not P1NP, are associated with cardiac alterations and 10-year outcome in an elderly community-based study

**DOI:** 10.1186/s12872-021-02138-8

**Published:** 2021-07-03

**Authors:** Jennifer M. T. A. Meessen, Giulia Cesaroni, Gian F. Mureddu, Alessandro Boccanelli, Ursula-Henrike Wienhues-Thelen, Peter Kastner, Luisa Ojeda-Fernandez, Deborah Novelli, Gianfranco Bazzoni, Maurizio Mangiavacchi, Nera Agabiti, Serge Masson, Lidia Staszewsky, Roberto Latini, on behalf of the PREDICTOR Investigators

**Affiliations:** 1 Department of Cardiovascular Medicine, Istituto di Ricerche Farmacologiche Mario Negri IRCCS, Milan, Italy; 2 Department of Epidemiology, Lazio Regional Health Service, ASL Roma 1, Rome, Italy; 3 Department of Cardiovascular Diseases, S Giovanni-Addolorata Hospital, Rome, Italy; 4 Casa di Cura Quisisana, Rome, Italy; 5 Roche Diagnostics GmbH, Penzberg, Germany; 6 Department of Biochemistry and Molecular Pharmacology, Istituto di Ricerche Farmacologiche Mario Negri IRCCS, Milan, Italy; 7 Centro Medico Specialistico UniSalus, Milan, Italy; 8 Roche Diagnostics International, Rotkreuz, Switzerland

**Keywords:** IGFBP7, GDF-15, P1NP, Cardiac remodelling, Community-dwelling elderly

## Abstract

**Background:**

Little is known about the clinical value of Insulin-like growth factor-binding protein-7 (IGFBP7), a cellular senescence marker, in an elderly general population with multiple co-morbidities and high prevalence of asymptomatic cardiovascular ventricular dysfunction. Inflammation and fibrosis are hallmarks of cardiac aging and remodelling. Therefore, we assessed the clinical performance of IGFBP7 and two other biomarkers reflecting these pathogenic pathways, the growth differentiation factor-15 (GFD-15) and amino-terminal propeptide of type I procollagen (P1NP), for their association with cardiac phenotypes and outcomes in the PREDICTOR study.

**Methods:**

2001 community-dwelling subjects aged 65–84 years who had undergone centrally-read echocardiography, were selected through administrative registries. Atrial fibrillation (AF) and 4 echocardiographic patterns were assessed: E/e’ (> 8), enlarged left atrial area, left ventricular hypertrophy (LVH) and reduced midwall circumference shortening (MFS). All-cause and cardiovascular mortality and hospitalization were recorded over a median follow-up of 10.6 years.

**Results:**

IGFBP7 and GDF-15, but not P1NP, were independently associated with prevalent AF and echocardiographic variables after adjusting for age and sex. After adjustment for clinical risk factors and cardiac patterns or NT-proBNP and hsTnT, both IGFBP7 and GDF-15 independently predicted all-cause mortality, hazard ratios 2.13[1.08–4.22] and 2.03[1.62–2.56] per unit increase of Ln-transformed markers, respectively.

**Conclusions:**

In a community-based elderly cohort**,** IGFBP7 and GDF-15 appear associated to cardiac alterations as well as to 10-year risk of all-cause mortality.

**Supplementary Information:**

The online version contains supplementary material available at 10.1186/s12872-021-02138-8.

## Background

Cardiovascular remodelling in the elderly is a complex phenomenon, which involves different pathways including hypertrophy, fibrosis, and cell death. Players are not limited to cardiac myocytes, but include fibroblasts, endothelial and smooth-muscle vascular cells. Several biomarkers have been made available over the recent years as possible readouts of the different processes involved [1].

In particular, insulin-like growth factor-binding protein-7 (IGFBP7) has been identified as a cellular senescence marker [2]. IGFBP7 is associated with cardiac structural and functional abnormalities, including hypertrophy, diastolic dysfunction and poor prognosis in patients with heart failure (HF) with and without atrial fibrillation (AF) [3–5]. These observations have been recently extended to insulin resistance and metabolic syndrome [6, 7]. Little is known on the clinical value of IGFBP7 in elderly general population with multiple co-morbidities and high prevalence of asymptomatic cardiovascular ventricular dysfunction.

Since inflammation and fibrosis are hallmarks of cardiac aging and remodelling, we compared the clinical performance of IGFBP7 with 2 other biomarkers reflecting these pathogenic pathways, i.e. the growth differentiation factor-15 (GDF-15) [8] and amino-terminal pro-peptide of type I procollagen (P1NP) [9]. GDF-15 is a member of the transforming growth factor-beta superfamily. Several studies have shown the strong prognostic value of GDF-15 in the general population [10, 11] and its relation with cardiac remodelling [12]. P1NP is a circulating marker of bone turnover, used to monitor evolution of osteoporosis [13]; P1NP was found altered in patients with HF indicating the activation of pro-fibrotic signalling and their prognostic relevance [14].

The PREDICTOR cohort of 2001 community-dwelling subjects aged 65–84 years was chosen since all subjects had undergone echocardiography, centrally read, and long-term outcome data were made available through administrative registries [15]. Taking the opportunity of the good characterization of the population and of the long-term follow-up, the present study was conducted with two complementary aims:

- To assess the relation of IGFBP7, GDF-15 and P1NP with atrial fibrillation, left ventricular hypertrophy (LVH), reduced mid-wall circumference fraction shortening (MFS), E/e’, and enlarged left atrium in an elderly community dwelling cohort;
- To estimate the long-term prognostic value for fatal and non-fatal clinical events.

## Methods

### Study population

PREDICTOR (Valutazione della PREvalenza di DIsfunzione Cardiaca asinTOmatica e di scompenso caRdiaco) was a cross-sectional, population-based study of the prevalence of asymptomatic LV dysfunction and heart failure (HF) in 2001 elderly people aged 65–84 years resident in the Lazio region of Italy.

The design of the PREDICTOR study has been described in detail [15]. A random sample of 5940 residents, 65–84 years old, from four cities (Rome, Civitavecchia, Frosinone, and Viterbo) in the Lazio region was identified based on the Regional Health Registry. Between June 2007 and January 2010, a total of 2001 subjects provided written informed consent. Participants were referred to eight cardiology centres in the Lazio region for clinical examination, blood tests, electrocardiography, comprehensive Doppler echocardiography and blood sampling to measure circulating biomarkers.

The Hospital Information System (HIS) provided data on hospitalizations occurred after the PREDICTOR baseline visit, while the Regional Mortality Registry provided mortality status with cause of death. Data from these two sources were linked through standardized methods based on a unique, anonymous, personal identifier, as reported elsewhere [16, 17]. A complete list of participating centres and investigators has been published [15]. Approval for this study was obtained from the local ethics committee.

Participants were followed-up from mid-2007 until 31st of December 2019 for all-cause mortality and hospitalization. Cause-specific mortality and hospitalization data was also available until 31 December, 2017. The ICD9 (mortality data) and ICD9CM (hospitalization data) codes 390–459 were classified as ‘cardiovascular’ and ICD9 code 428.x was classified as heart failure [18].

## Circulating biomarkers

Venous blood samples from fasted subjects were collected with participants resting in the supine position for at least 15 min. Blood samples were collected in tubes containing ethylendiamine tetraacetic acid tripotassium salt (EDTA). Blood was centrifuged at 2000 g at 4 °C within 10 min and aliquots of plasma were immediately frozen and subsequently transported on dry ice to a central laboratory. Samples were stored at − 70 °C until they were assayed. Plasma concentrations of all biomarkers were assayed in a central laboratory by personnel blinded to the identity of each sample. NT-proBNP, hs-cTnT, GDF-15, P1NP, were measured by electrochemiluminescence immunoassay using commercial reagents (cobas Elecsys® 2010, Roche Diagnostics GmbH, Mannheim). The cardiac biomarkers, hs-cTnT and NT-proBNP, were used as benchmark.

IGFBP7 was measured using a preclinical research-use only assay on an automated platform (Roche Diagnostics GmbH, Penzberg, Germany). The detection method for IGFBP7 was a sandwich immunoassay developed on the Elecsys® platform for electrochemiluminescence detection (Roche Diagnostics GmbH, Mannheim, Germany). Mouse monoclonal antibodies were generated and screened for specific detection of IGFBP7. Precision within-run coefficient of variation for IGFBP7 was 2%, the limit of detection was 0.01 ng/mL.

## Echocardiography and cardiac phenotype

Color Doppler echocardiography was performed in participating centres using commercially available machines, according to a predefined acquisition protocol, and centrally read [15]. Details on echocardiographic methods used for LV function and mass, and staging of heart failure have been reported [8, 12], and are summarized for convenience in Additional file 1: Supplemental Material.

Atrial fibrillation was diagnosed at 12-lead ECG at study entry.

The following cardiac phenotypes were defined based on echocardiographic exam at study entry:

- Left ventricular hypertrophy (LVH): sex-specific LVH was defined as Left Ventricular Mass/Body Surface Area > 95 g/m^2^ for women and > 115 g/m^2^ for men.
- Mid-wall circumference fraction shortening (MFS): Reduced MFS was defined as < 15%.
- Diastolic dysfunction defined as E/e’ > 8. [19] 
- Enlarged left atrium, defined as left atrial area (LAA) > 20 cm^2^/m^2^ based on the recommendations of the American society of echocardiography) [20].

In addition, 1715 participants were matched with data from the Hospital Information System (HIS) until 31 December 2019, allowing a median follow-up of 10.6 years [2 months to 12.5 years] for all-cause mortality, but of 2 years less for cause-specific mortality. The following long term outcomes were assessed:

- All-cause mortality
- Cardiovascular mortality, (data available until 31/12/17);
- All-cause hospitalization
- Cardiovascular hospitalization

### Statistical methods

Baseline characteristics are reported by means of descriptive statistics. Categorical variables are presented as proportions. Normally distributed continuous variables are expressed as mean (SD) and compared by means of ANOVA while non-parametric variables are expressed as median [Q1–Q3] and compared by Kruskal–Wallis. Proportions were compared by means of Fisher’s exact test. The correlations between the biomarkers (IGFBP7, GDF-15, P1NP, hs-cTnT and NT-proBNP) were assessed by means of Spearman Rank non-parametric test. Binary logistic univariate and multivariable regression models adjusted for age and sex were used to assess the association between ln-transformed biomarkers and the cardiac phenotypes. P1NP was not included in the analysis due to its lack of relation with clinical outcomes. Kaplan–Meier curves were constructed for the tertiles of IGFBP7 and GDF-15. Cox-proportional hazard models were used to assess predictive value of ln-transformed IGFBP7 or GDF-15 for clinical outcomes, adjusted for those variables found to different between tertiles of biomarker univariate. SPSS v26.0 (IBM SPSS, Armonk, NY, USA) was used for statistical analysis. A *p* value of < 0.05 was considered statistically significant.

## Results

### Clinical correlates of plasma concentrations of the 3 circulating biomarkers

The demographic, clinical and echocardiographic characteristics of participants according to tertiles of IGFBP7, GDF-15 and P1NP concentrations are shown in Tables 1, 2 and 3. The median [Q1–Q3] concentrations of IGFBP7, GFD-15 and P1NP in the overall population were 166 [151–184] ng/mL, 1468 [1168–1984] pg/mL and 35.2 [26.3–46.0] ng/mL, respectively. Both GDF-15 and P1NP were above the reference normal values. IGFBP7 and GDF-15 were correlated with each other (r = 0.474) and with hsTnT (IGFBP7: r = 0.394; GDF15: r = 0.433) and NT-proBNP (IGFBP7: r = 0.337; GDF15: r = 0.305), all correlations had *p* < 0.0001.

**Table 1:** Demographic, clinical and echocardiographic characteristics according to tertiles of IGF BP 7

		Total	IGFBP7 (ng/mL)			*p*
			Tertile 1	Tertile 2	Tertile 3	
		N = 1913	N = 631	N = 632	N = 650	
IGFBP7	Median [IQR]	165.5 [150.7–183.6]	145.4 [138.0–150.6]	165.1 [160.2–170.5]	193.9 [182.8–210.3]	–
	Range	92.4–617.8	92.4–155.2	155.3–175.8	175.9–617.8	
Age, years	Mean ± SD	72.7 ± 5.0	70.8 ± 4.1	72.2 ± 4.5	75.2 ± 5.2	3.4 × 10^−61^
Females	N (%)	925 (48.4)	366 (58.0)	309 (48.9)	250 (38.5)	2.2 × 10^−11^
BMI, kg/m^2^	Mean ± SD	26.5 ± 4.2	26.2 ± 4.0	26.7 ± 4.1	26.5 ± 4.3	0.084
BSA, m^2^	Mean ± SD	1.80 ± 0.19	1.77 ± 0.18	1.82 ± 0.20	1.83 ± 0.18	6.4 × 10^−9^
Serum creatinine, mg/dL^−1^	Mean ± SD	0.96 ± 0.26	0.85 ± 0.17	0.93 ± 0.19	1.10 ± 0.33	1.6 × 10^−71^
eGFR, mL/min/1.73m^2^	Mean ± SD	69.6 ± 21.1	75.6 ± 19.8	72.2 ± 21.5	61.0 ± 19.0	1.4 × 10^−38^
CKD (eGFR < 60)	N (%)	632 (33.4%)	123 (19.6%)	176 (27.9%)	333 (52.2%)	3.6 × 10^−36^
*Clinical history*						
Hypertension	N (%)	1132 (59.2)	357 (56.6)	375 (59.3)	400 (61.5)	0.195
Diabetes mellitus	N (%)	313 (16.5)	91 (14.6)	91 (14.4)	131 (20.2)	0.006
Smoking	N (%)	252 (13.2)	88 (13.9)	83 (13.2)	81 (12.5)	4.1 × 10^−7^
Alcohol use	N (%)	1128 (59.0)	367 (58.2)	382 (60.5)	379 (58.3)	0.628
Dyslipidemia	N (%)	828 (44.3)	302 (49.2)	268 (43.1)	258 (40.8)	0.009
Angina pectoris	N (%)	121 (6.3)	41 (6.5)	41 (6.5)	39 (6.0)	0.916
Myocardial infarction	N (%)	116 (6.1)	34 (5.4)	28 (4.5)	54 (8.4)	0.010
Atrial fibrillation	N (%)	132 (6.9)	28 (4.4)	35 (5.5)	69 (10.6)	1.9 × 10^−5^
Heart failure	N (%)	114 (6.3)	18 (3.0)	35 (5.7)	61 (10.0)	2.0 × 10^−6^
*AHA/ACC class*						
Normal	N (%)	242 (12.7)	104 (16.5)	85 (13.4)	53 (8.2)	1.8 × 10^−7^
A	N (%)	444 (23.2)	148 (23.5)	139 (22.0)	157 (24.2)	
B	N (%)	1113 (58.2)	361 (57.2)	373 (59.0)	379 (58.3)	
C	N (%)	114 (6.0)	18 (2.9)	35 (5.5)	61 (9.4)	
COPD	N (%)	171 (8.9)	48 (7.6)	56 (8.9)	67 (10.3)	0.237
*Circulating biomarkers*						
GDF-15, pg/mL	Median [IQR]	1468 [1168–1984]	1250 [1021–1527]	1405 [1168–1761]	1915 [1445–2603]	3.7 × 10^−82^
P1NP, ng/mL	Median [IQR]	35.2 [26.3–46.0]	32.6 [24.5–42.6]	34.9 [26.0–45.5]	37.2 [27.5–51.3]	2.9 × 10^−7^
hs cTnT, ng/L	Median [IQR]	5.5 [3.0–9.5]	3.5 [3.0–6.3]	5.0 [3.0–8.2]	8.1 [4.9–13.7]	5.7 × 10^−58^
NT-proBNP, ng/L	Median [IQR]	92 [47–186]	63 [36–123]	81 [45–151]	142 [69–293]	8.9 × 10^−42^
*Echocardiography*						
LVEF, % (N = 1858)	Mean ± SD	66.3 ± 7.2	66.9 ± 6.2	66.7 ± 6.9	65.2 ± 8.3	8.1 × 10^−5^
LAA-, cm^2^ (N = 1332)	Mean ± SD	11.1 ± 4.1	10.4 ± 3.6	10.9 ± 3.5	11.9 ± 4.9	8.1 × 10^−7^
LV mass / BSA, g/m^2^ (N = 1489)	Mean ± SD	91.9 ± 23.3	87.4 ± 19.5	92.7 ± 23.8	95.8 ± 25.4	2.5 × 10^−8^
LVH (N = 1853)	N (%)	373 (24.4)	99 (18.9)	127 (25.1)	147 (29.3)	4.9 × 10^−4^
MFS reduced (N = 1470)	N (%)	448 (31.9)	136 (28.6)	137 (29.6)	175 (37.5)	0.006
E/e’ > 8 (N = 1801)	N (%)	765 (44.4)	216 (38.0)	261 (45.5)	288 (49.7)	3.2 × 10^−4^
Enlarged LA-area (N = 1332)	N (%)	41 (3.2)	7 (1.7)	8 (1.8)	26 (6.2)	1.2 × 10^−4^
*Outcomes (N* = *1715)*						
All-cause mortality	N (%)	491 (30.0)	104 (19.7)	130 (23.8)	257 (45.7)	4.9 × 10^−23^
CV mortality	N (%)	115 (6.0)	19 (3.0)	24 (3.8)	72 (11.1)	1.6 × 10^−10^
Hospitalization	N (%)	1297 (79.3)	392 (74.4)	439 (80.3)	466 (82.9)	0.002
CV hospitalization	N (%)	583 (35.6)	143 (27.1)	210 (38.4)	230 (40.9)	3.0 × 10^−6^
HF hospitalization	N (%)	168 (10.3)	25 (4.7)	50 (9.1)	93 (16.3)	6.7 × 10^−10^

BMI, body mass index; BSA, body surface area; CKD, chronic kidney disease defined as eGFR < 60; CV, cardiovascular; COPD, chronic obstructive pulmonary disease; E/e ‘ > 8 vs <  = 8; eGFR, estimated glomerular filtration rate; GDF-15, growth differentiation factor-15; hs cTnT, high sensitivity cardiac troponin T; IGFBP7, insulin grow factor binding protein; LAA, left atrial area, enlarged if > 20 cm^2^; LVEF, left ventricular ejection fraction; LVH: Left ventricular hypertrophy defined as LV mass/BSA > 95 g/m^2^ for women and > 115 g/m^2^ for men; MFS, midwall circumference fraction shortening, reduced if MFS < 15%; NTproBNP, N-terminal probrain natriuretic peptide; P1NP, amino-terminal propeptide of type I procollagen. Continuous data is presented either as mean ± SD or median [IQR]

**Table 2:** Demographic, clinical and echocardiographic characteristics according to tertiles of GDF-15

		Total	GDF-15 (pg/mL)			*p*
			Tertile 1	Tertile 2	Tertile 3	
		N = 1907	N = 642	N = 625	N = 640	
GDF-15	Median [IQR]	1468 [1168–1984]	1059 [932–1168]	1460 [1368–1605]	2267 [1960–2991]	–
	Range	592–13,015	592–1272	1273–1760	1762–13,015	
Age, years	Mean ± SD	72.9 ± 5.0	70.6 ± 3.8	72.9 ± 4.7	75.1 ± 5.3	5.6 × 10^−66^
Females	N (%)	962 (48.3%)	393 (59.6%)	316 (48.2%)	253 (37.4%)	4.9 × 10^−15^
BMI, kg/m^2^	Mean ± SD	26.5 ± 4.2	26.2 ± 4.2	26.7 ± 4.1	26.5 ± 4.2	0.105
BSA, m^2^	Mean ± SD	1.80 ± 0.19	1.78 ± 0.19	1.81 ± 0.19	1.82 ± 0.18	1.8 × 10^−4^
Serum creatinine, mg/dL^−1^	Mean ± SD	0.96 ± 0.27	0.87 ± 0.20	0.94 ± 0.21	1.07 ± 0.33	1.5 × 10^−46^
eGFR, mL/min/1.73m^2^	Mean ± SD	69.3 ± 21.0	75.2 ± 21.3	70.0 ± 19.4	62.9 ± 20.6	2.9 × 10^−26^
CKD (eGFR < 60)	N (%)	671 (34.0%)	141 (12.5%)	209 (32.3%)	321 (48.1%)	1.0 × 10^−23^
*Clinical history*						
Hypertension	N (%)	1183 (59.4%)	385 (58.4%)	394 (60.2%)	404 (59.8%)	0.798
Diabetes mellitus	N (%)	330 (16.7%)	47 (7.2%)	94 (14.5%)	189 (28.0%)	6.2 × 10^−24^
Smoking	N (%)	259 (13.0%)	49 (7.4%)	86 (13.1%)	124 (18.4)%	7.3 × 10^−15^
Alcohol use	N (%)	1170 (58.8%)	399 (60.5%)	386 (58.9%)	385 (57.0%)	0.427
Dyslipidaemia	N (%)	854 (43.9%)	289 (44.6%)	300 (46.9%)	265 (40.2%)	0.046
Angina pectoris	N (%)	123 (6.2%)	24 (3.6%)	35 (5.3%)	64 (9.5%)	3.2 × 10^−5^
Myocardial infarction	N (%)	122 (6.2%)	16 (2.4%)	34 (5.2%)	72 (10.7%)	1.4 × 10^−9^
Atrial fibrillation	N (%)	153 (7.7%)	29 (44.4%)	44 (6.7%)	80 (11.8%)	1.0 × 10^−6^
Heart failure	N (%)	127 (6.7%)	15 (2.4%)	35 (5.6%)	77 (12.3%)	6.0 × 10^−12^
*AHA/ACC class*						
Normal	N (%)	249 (12.5%)	112 (17.0%)	82 (12.5%)	55 (8.1%)	2.2 × 10^−12^
A	N (%)	466 (23.4%)	158 (24.0%)	153 (23.4%)	155 (22.9%)	
B	N (%)	1148 (57.7%)	374 (56.8%)	385 (58.8%)	389 (57.5%)	
C	N (%)	127 (6.4%)	15 (2.3%)	35 (5.3%)	77 (11.4%)	
COPD	N (%)	178 (8.9%)	34 (5.2%)	67 (10.2%)	77 (11.4%)	1.3 × 10^−4^
*Circulating biomarkers*						
IGFBP7, ng/mL	Median [IQR]	166 [151–184]	155 [144–167]	166 [153–179]	182 [163–203]	4.0 × 10^−80^
P1NP, ng/mL	Median [IQR]	35.2 [26.3–46.0]	35.1 [26.8–44.8]	35.4 [26.6–45.1]	35.1 [25.7–49.3]	0.756
hs cTnT, ng/L	Median [IQR]	5.5 [3.0–9.5]	3.3 [3.0–5.9]	5.6 [3.0–9.0]	8.5 [4.9–14.1]	2.8 × 10^−69^
NT-proBNP, ng/L	Median [IQR]	92 [47–186]	66 [37–122]	90 [47–180]	135 [66–299]	1.1 × 10^−32^
*Echocardiography*						
LVEF, % (N = 1858)	Mean ± SD	66.1 ± 7.4	67.0 ± 6.2	66.5 ± 6.8	64.6 ± 8.8	4.4 × 10^−8^
LAA-, cm^2^ (N = 1332)	Mean ± SD	11.2 ± 4.4	10.7 ± 3.8	11.1 ± 4.3	12.0 ± 4.9	1.9 × 10^−5^
LV mass / BSA, g/m^2^ (N = 1489)	Mean ± SD	92.2 ± 23.2	87.9 ± 20.3	91.1 ± 22.0	98.1 ± 26.3	2.4 × 10^−12^
LVH (N = 1853)	N (%)	396 (24.8%)	119 (21.0%)	119 (22.6%)	158 (31.5%)	1.2 × 10^−4^
MFS reduced (N = 1470)	N (%)	471 (32.2%)	120 (23.4%)	159 (32.6%)	195 (41.4%)	1.6 × 10^−8^
E/e’ > 8 (N = 1801)	N (%)	801 (44.7%)	239 (39.6%)	262 (43.9%)	300 (50.8%)	0.001
Enlarged LA-area (N = 1332)	N (%)	54 (4.1%)	10 (2.1%)	18 (4.1%)	26 (6.2%)	0.008
*Outcomes (N* = *1715)*						
All-cause mortality	N (%)	526 (30.8%)	81 (14.8%)	139 (24.6%)	306 (51.4%)	5.6 × 10^−43^
CV mortality	N (%)	125 (6.3%)	14 (2.1%)	33 (5.0%)	78 (11.5%)	3.4 × 10^−12^
Hospitalization	N (%)	1360 (79.7%)	410 (75.0%)	448 (79.3%)	502 (84.4%)	4.0 × 10^−4^
CV hospitalization	N (%)	613 (35.9%)	153 (28.0%)	192 (34.0%)	268 (45.0%)	7.4 × 10^−9^
HF hospitalization	N (%)	184 (10.8%)	26 (4.8%)	62 (11.0%)	96 (16.1%)	4.5 × 10^−9^

BMI, body mass index; BSA, body surface area; CKD, chronic kidney disease defined as eGFR < 60; CV, cardiovascular; COPD, chronic obstructive pulmonary disease; E/e ‘ > 8 vs <  = 8; eGFR, estimated glomerular filtration rate; GDF-15, growth differentiation factor-15; hs cTnT, high sensitivity cardiac troponin T; IGFBP7, insulin grow factor binding protein; LAA, left atrial area, enlarged if > 20 cm^2^; LVEF, left ventricular ejection fraction; LVH: Left ventricular hypertrophy defined as LV mass/BSA > 95 g/m^2^ for women and > 115 g/m^2^ for men; MFS, midwall circumference fraction shortening, reduced if MFS < 15%; NTproBNP, N-terminal probrain natriuretic peptide; P1NP, amino-terminal propeptide of type I procollagen. Continuous data is presented either as mean ± SD or median [IQR]

**Table 3:** Demographic, clinical and echocardiographic characteristics of the participants according to tertiles of P1NP

		Total	P1NP			*p*
			Tertile 1	Tertile 2	Tertile 3	
		N = 1991	N = 656	N = 659	N = 676	
P1NP	Median [IQR]	35.2 [26.3–46.0]	23.0 [19.2–26.2]	35.0 [31.6–37.7]	52.4 [45.8–64.2]	
	Range	6.34–1200	6.34–29.2	29.3–41.1	41.2–1200	
Age, years	Mean ± SD	72.9 ± 5.0	72.63 ± 4.8	72.7 ± 5.0	73.3 ± 5.2	0.035
Females	N (%)	962 (48.3%)	253 (38.6%)	304 (46.1%)	405 (59.9%)	2.5 × 10^−14^
BMI, kg/m^2^	Mean ± SD	26.5 ± 4.2	26.5 ± 3.9	26.7 ± 4.3	26.3 ± 4.2	0.192
BSA, m^2^	Mean ± SD	1.80 ± 0.19	1.82 ± 0.18	1.81 ± 0.20	1.77 ± 0.18	2.0 × 10^−6^
Serum creatinine, mg/dL^−1^	Mean ± SD	0.96 ± 0.27	0.96 ± 0.21	0.96 ± 0.26	0.96 ± 0.32	0.947
eGFR, mL/min/1.73m^2^	Mean ± SD	69.3 ± 21.0	70.7 ± 20.4	70.6 ± 22.3	66.7 ± 20.2	3.8 × 10^−4^
CKD (eGFR < 60)	N (%)	671 (34.0%)	206 (31.5%)	212 (32.6%)	253 (37.9%)	0.030
*Clinical history*						
Hypertension	N (%)	1183 (59.4%)	387 (59.0%)	388 (58.9%)	408 (60.4%)	0.829
Diabetes mellitus	N (%)	330 (16.7%)	148 (22.7%)	98 (15.0%)	84 (12.5%)	2.0 × 10^−6^
Smoking	N (%)	259 (13.0%)	89 (13.6%)	93 (14.1%)	77 (11.4%)	1.2 × 10^−4^
Alcohol use	N (%)	1171 (58.8%)	405 (61.8%)	401 (60.8%)	365 (54.0%)	0.006
Dyslipidaemia	N (%)	854 (43.9%)	290 (45.3%)	284 (44.3%)	280 (42.0%)	0.474
Angina pectoris	N (%)	123 (6.2%)	47 (7.2%)	38 (5.8%)	38 (5.6%)	0.437
Myocardial infarction	N (%)	122 (6.2%)	42 (6.4%)	37 (5.7%)	43 (6.4%)	0.792
Atrial fibrillation	N (%)	153 (7.7%)	45 (6.9%)	55 (8.3%)	53 (7.8%)	0.589
COPD	N (%)	178 (8.9%)	67 (10.2%)	56 (8.5%)	55 (8.1%)	0.368
Heart failure	N (%)	127 (6.7%)	38 (6.1%)	41 (6.6%)	48 (7.5%)	0.605
*AHA/ACC class*						
Normal	N (%)	250 (12.6%)	78 (11.9%)	85 (12.9%)	87 (12.9%)	0.103
A	N (%)	466 (23.4%)	152 (23.2%)	178 (27.0%)	136 (20.1%)	
B	N (%)	1148 (57.7%)	388 (59.1%)	355 (53.9%)	405 (59.9%)	
C	N (%)	127 (6.4%)	38 (5.8%)	41 (6.2%)	48 (7.1%)	
*Circulating biomarkers*						
IGFBP7, ng/mL	Median [IQR]	166 [151–184]	163 [149–179]	164 [149–182]	170 [154–192]	2.2 × 10^−7^
GDF-15, pg/mL	Median [IQR]	1468 [1168–1984]	1478 [1160–2046]	1435 [1160–1878]	1499 [1186–2070]	0.058
hs cTnT, ng/L	Median [IQR]	5.5 [3.0–9.5]	6.0 [3.0–9.8]	5.2 [3.0–8.6]	5.4 [3.0–10.4]	0.147
NT-proBNP, ng/L	Median [IQR]	92 [47–186]	85 [43–167]	87 [45–169]	112 [53–219]	9.0 × 10^−6^
*Echocardiography and outcomes*						
LVEF, % N = 1858)	Mean ± SD	66.1 ± 7.4	66.0 ± 7.6	66.2 ± 7.1	65.9 ± 7.5	0.803
LAA-, cm^2^ (N = 1332)	Mean ± SD	11.2 ± 4.4	11.1 ± 4.1	11.0 ± 4.3	11.6 ± 4.6	0.105
LV mass / BSA, g/m^2^ (N = 1489)	Mean ± SD	92.2 ± 23.2	94.2 ± 24.5	91.5 ± 23.2	90.9 ± 21.8	0.045
LVH (N = 1853)	N (%)	396 (24.8%)	128 (23.9%)	123 (23.6%)	145 (26.9%)	0.400
MFS reduced (N = 1470)	N (%)	471 (32.2%)	170 (34.2%)	144 (30.4%)	157 (31.8%)	0.435
E/e’ > 8 (N = 1801)	N (%)	801 (44.7%)	267 (45.4%)	239 (40.2%)	295 (48.4%)	0.016
Enlarged LA-area (N = 1332)	N (%)	54 (4.1%)	16 (3.7%)	15 (3.4%)	23 (5.1%)	0.413
*Outcomes (N* = *1715)*						
All-cause mortality	N (%)	526 (30.8%)	169 (30.0%)	159 (28.3%)	198 (34.0%)	0.098
CV mortality	N (%)	125 (6.3%)	38 (5.8%)	35 (5.3%)	52 (7.7%)	0.165
Hospitalization	N (%)	1360 (79.7%)	455 (80.8%)	443 (78.8%)	462 (79.4%)	0.693
CV hospitalization	N (%)	613 (35.9%)	217 (38.5%)	188 (33.5%)	208 (35.7%)	0.204
HF hospitalization	N (%)	184 (10.8%)	60 (10.7%)	50 (8.9%)	74 (12.7%)	0.114

BMI, body mass index; BSA, body surface area; CKD, chronic kidney disease defined as eGFR < 60; CV, cardiovascular; COPD, chronic obstructive pulmonary disease; E/e ‘ > 8 vs <  = 8; eGFR, estimated glomerular filtration rate; GDF-15, growth differentiation factor-15; hs cTnT, high sensitivity cardiac troponin T; IGFBP7, insulin grow factor binding protein; LAA, left atrial area, enlarged if > 20 cm^2^; LVEF, left ventricular ejection fraction; LVH: Left ventricular hypertrophy defined as LV mass/BSA > 95 g/m^2^ for women and > 115 g/m^2^ for men; MFS, midwall circumference fraction shortening, reduced if MFS < 15%; NTproBNP, N-terminal probrain natriuretic peptide; P1NP, amino-terminal propeptide of type I procollagen. Continuous data is presented either as mean ± SD or median [IQR]

Subjects with IGFBP7 in the tertile 3 were older, less frequently females or smokers, and with decreased renal function and more often cardiovascular risk factors and disorders. Tertile 3 of IGFBP7 was associated with higher concentrations of all circulating biomarkers, in particular GDF-15, hsTnT and NT-proBNP. In multiple linear regression analyses the strongest variables independently associated with higher concentrations of IGFBP7 were creatinine, age and heart failure (all *p* < 0.0001).

Tertiles 2 and 3 of GDF-15 were older, had significantly higher creatinine levels as well as a larger proportion males and smokers. Higher GDF-15 was associated with more patients with diabetes, angina pectoris, myocardial infarction, atrial fibrillation and COPD. Tertile 3 of GDF-15 was associated with higher concentrations of all circulating biomarkers except for P1NP. In addition, higher GDF-15 were associated with higher proportions of altered echocardiographic patterns (Table 2). Age, creatinine, diabetes and atrial fibrillation were strongly, independently associated with GDF-15 in multivariate regression model (all *p* < 0.0001).

P1NP was higher in females, in non-diabetics and in non-smokers (Table 3). Upon including these variables in multivariable regression, only diabetes, sex creatinine and age were associated with higher P1NP.

### Relationship between concentrations of the 3 biomarkers and echocardiographic variables

Atrial fibrillation and four echocardiographic patterns were dichotomized by presence vs absence: increased E/e’, enlarged LAA, left ventricular hypertrophy, and reduced mid-wall circumference fraction shortening. Ln-transformed IGFBP7 was associated with all variables after adjustment for age and sex. All echocardiographic characteristics, except for enlarged LAA, were independently associated to Ln-transformed GDF-15, whereas Ln-transformed P1NP was not associated with any echocardiographic characteristic (Table 4).

**Table 4:** Results of logistic regression models

Dependent variable	Univariate			Adjusted by age and sex			Adjusted by age, sex and hypertension		
	OR	(95% CI)	*p*	OR	(95% CI)	*p*	OR	(95% CI)	*p*
*IGFBP7*									
Atrial fibrillation	8.87	3.71–21.20	9.1 × 10^−7^	6.70	2.59–17.32	8.8 × 10^−5^	6.79	2.62–17.60	8.0 × 10^−5^
E/e’	0.23	0.13–0.41	7.0 × 10^−7^	0.33	0.17–0.64	0.001	0.34	0.18–0.64	0.001
Enlarged LAA	21.64	5.77–81.21	5.0 × 10^−−6^	12.68	3.01–53.42	0.001	12.72	3.01–53.86	0.001
LVH	4.96	2.54–9.69	3.0 × 10^−6^	4.67	2.24–9.73	3.9 × 10^−5^	4.51	2.15–9.48	6.8 × 10^−5^
MFS	4.24	2.21–8.16	1.5 × 10^−5^	2.52	1.25–5.07	0.010	2.44	1.21–4.93	0.013
*GDF–15*									
Atrial fibrillation	2.79	2.01–3.87	7.2 × 10^−10^	2.52	1.77–3.57	2.7 × 10^−7^	2.51	1.77–3.57	3.0 × 10^−7^
E/e’	0.62	0.50–0.78	2.3 × 10^−5^	0.72	0.56–0.91	0.006	0.71	0.56–0.90	0.004
Enlarged LAA	2.45	1.45–4.13	0.001	1.67	0.90–3.07	0.103	1.70	0.92–3.14	0.091
LVH	1.70	1.33–2.19	3.4 × 10^−5^	1.67	1.27–2.19	2.8 × 10^−4^	1.72	1.30–2.27	1.4 × 10^−4^
MFS	2.18	1.70–2.81	1.2 × 10^−9^	1.85	1.41–2.42	8.0 × 10^−6^	1.89	1.44–2.48	4.0 × 10^−6^
*P1NP*									
Atrial fibrillation	1.27	0.89–1.82	0.189	1.19	0.83–1.72	0.337	1.20	0.83–1.72	0.334
E/e’	0.88	0.72–1.09	0.238	0.99	0.81–1.24	0.994	1.00	0.81–1.25	0.977
Enlarged LAA	1.44	0.80–2.57	0.222	1.32	0.74–2.36	0.354	1.31	0.73–2.34	0.369
LVH	1.12	0.87–1.44	0.376	0.96	0.74–1.25	0.777	0.96	0.74–1.25	0.761
MFS	0.92	0.72–1.17	0.494	0.90	0.71–1.15	0.416	0.91	0.71–1.16	0.445

Logistic univariate and multivariate regression analyses with cardiac phenotype (dependent) and Ln-transformed IGFBP7, GDF-15 and P1NP

E/e ‘ > 8 vs <  = 8; GDF-15, growth differentiation factor-15; IGFBP7, insulin grow factor binding protein; P1NP, amino-terminal propeptide of type I procollagen. LAA, left atrial area, enlarged if > 20; LVH: Left ventricular hypertrophy defined as LV mass/BSA > 95 g/m^2^ for women and > 115 g/m^2^ for men; MFS, midwall circumference fraction shortening, reduced if MFS < 15%

### Prognostic value of IGFBP7 and GDF-15

During a median of 10.6 years of follow-up, 526 patients (26.3%) died, and 1365 (68.2%) were admitted to hospital for any reason. Both IGFBP7 and GDF-15 had significantly higher all-cause and cardiovascular mortality in their highest tertiles as well as a significantly increased all-cause and cardiovascular hospitalization. On the contrary, P1NP appeared totally unrelated to study outcomes (data not shown).

**Fig. 1:**
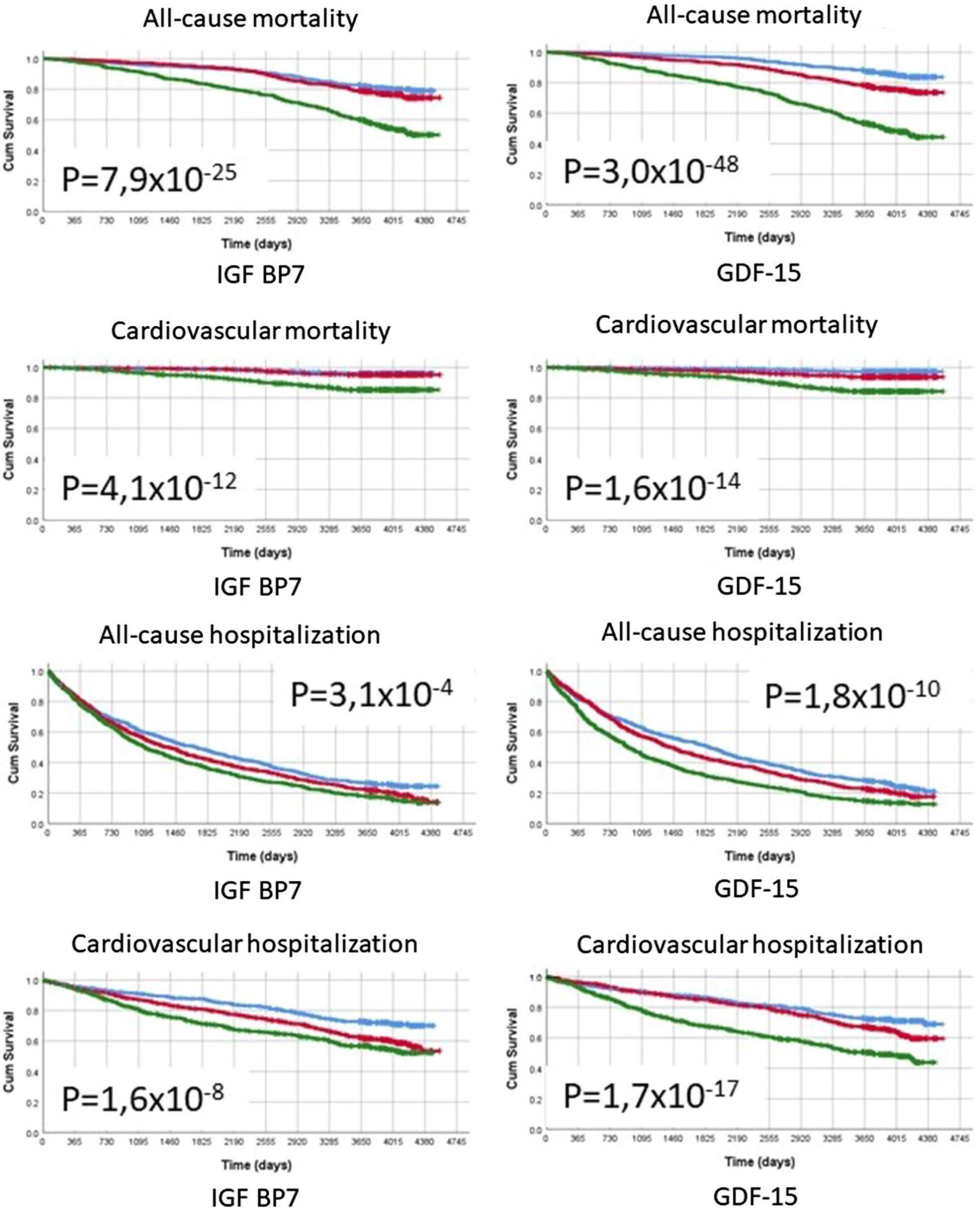
Kaplan–Meier curves for: all-cause and cardiovascular mortality, all-cause-, cardiovascular- and heart failure hospitalization split by tertiles of IGFBP7 and GDF-15. *p* value for log-rank test for the comparison of Kaplan–Meier estimates. Blue—lowest tertile, red- middle tertile, green—highest tertile

Figure 1 shows the Kaplan–Meier survival curves for these outcomes (all-cause and cardiovascular mortality and hospitalization). In general, patients in tertile 3 had worse outcomes, as evident from Tables 1 and 2. In univariate Cox analyses, increased concentrations of IGFBP7 and GDF-15 predicted mortality and hospitalization either all-cause or cardiovascular (Table 5). After adjustment for clinical variables, the association with all-cause and cardiovascular mortality remained significant for both IGFBP7 and GFD-15: HR 2.13[1.08–4.22] and 2.03[1.62–2.56] per unit increase of Ln-transformed markers, respectively. Upon adjusting for echocardiographic variables (e.g. MFS and enlarged LAA) or biomarkers (e.g. NT-proBNP and hsTnT), IGFBP7 and GDF-15 no longer independently predicted hospitalizations but only mortality.

**Table 5:** Results of Cox proportional hazard regression models

	N	All-cause mortality		CV mortality		Hospitalization		CV hospitalization	
		HR[95CI]	*p*	HR[95CI]	*p*	HR[95CI]	*p*	HR[95CI]	*p*
*IGFBP7*									
Univariate	1634	17.17 [11.31–26.05]	1.1 × 10^−40^	31.7 [15.06–66.87]	9.8 × 10^−20^	2.31 [1.66–3.21]	7.5 × 10^−7^	4.48 [2.89–6.96]	2.5 × 10^−11^
Multivariable	1486	5.20 [2.79–9.68]	2.0 × 10^−7^	7.73 [2.28–26.26]	0.001	1.50 [0.99–2.28]	0.057	2.26 [1.27–4.01]	0.006
Multivariable + echo	809	4.25 [1.81–9.99]	0.001	2.52 [0.47–13.39]	0.279	1.11 [0.61–2.01]	0.733	1.65 [0.76–3.57]	0.204
Multivariable + biomarkers	1486	2.13 [1.08–4.22]	0.029	1.97 [0.51–7.66]	0.329	0.94 [0.60–1.48]	0.794	1.04 [0.55–1.94]	0.911
*GDF-15*									
Univariate	1705	3.44 [2.95–4.02]	4.0 × 10^−56^	3.96 [2.92–5.36]	6.0 × 10^−19^	1.60 [1.42–1.80]	3.0 × 10^−14^	1.91 [1.63–2.24]	2.3 × 10^−15^
Multivariable	1621	2.58 [2.09–3.20]	2.5 × 10^−18^	2.84 [1.82–4.43]	4.0 × 10^−6^	1.32 [1.13–1.53]	3.3 × 10^−4^	1.50 [1.21–1.84]	1.2 × 10^−4^
Multivariable + echo	843	2.50 [1.82–3.44]	1.9 × 10^−8^	1.89 [0.96–3.73]	0.067	1.42 [1.15–1.77]	0.001	1.44 [1.09–1.92]	0.011
Multivariable + biomarkers	1621	2.03 [1.62–2.56]	1.5 × 10^−9^	1.75 [1.06–2.88]	0.028	1.17 [0.99–1.37]	0.059	1.22 [0.98–1.53]	0.079

Cox proportional hazard models for all-cause mortality or hospital admission for cardiovascular reason. HR [95%CI] for Ln-transformed concentration of the biomarker

For IGFBP7 the multivariable regression analysis was corrected for age, sex, eGFR, diabetes, smoking, dyslipidaemia, MI, AF, heart failure as identified in Table 1

For GDF-15 the multivariable regression analysis was corrected for age, sex, eGFR, diabetes, smoking, dyslipidaemia, angina, MI, AF and COPD, as identified in Table 2

Echo: included MFS and enlarged left atrial area in the regression analyses

Biomarkers: included log-transformed hsTnT and log-transformed NTproBNP in the regression analysis

In addition, IGFBP7 and more so GDF-15 predicted cancer mortality (147 events, 27.9% of all deaths). Death rates for cancer in the upper tertile of GDF-15 and IGFBP7 were 11.5% and 9.7%, compared to 3.9% and 6.0% in the lower tertile (*p* < 0.0001 and *p* = 0.011, respectively). In addition, Cox proportional hazard regression analyses, adjusted for age, sex, systolic blood pressure, diabetes, COPD, alcohol consumption, atrial fibrillation, heart failure, smoking, dyslipidemia, history of ischemic heart disease, LVEF and LV mass/BSA, showed significant results for both IGFBP7 (HR:3.97 [95% CI 1.16–13.58], *p* = 0.028) and GDF15 (HR: 2.04 [1.30–3.19], *p* = 0.002).

## Discussion

In a cross-sectional epidemiological study including almost 2000 community-dwelling elderly persons (65–84 years) living in the region of Rome, Italy, and followed up for 10 years, the novel biomarker IGFBP7 was found to be associated with cardiac characteristics related to aging, such as LV hypertrophy and mild LV systolic dysfunction. Atrial fibrillation, enlarged LAA and E/e’ > 8 were also associated with higher concentrations of IGFBP7. IGFBP7 was also independently associated with mortality, all-cause as well as cardiovascular.

Similarly, GDF-15 was found to be associated with echocardiographic variables such as LVH and MFS, atrial fibrillation and E/e’ > 8. In addition, after adjusting for clinical characteristics, GDF-15 was predictive for mortality (both all-cause and cardiovascular). On the other side, P1NP, a marker of fibrosis, did not show any association with cardiac phenotypes or with outcomes (data not shown). If other circulating markers of collagen turnover, such as PIIINP, PICP had been assayed, more encouraging results may have been obtained [21]. In general, the concentrations of IGFBP7 and GDF-15 were lower than those reported in other studies, focused on patients with cardiovascular disease. Indeed, in the present elderly cohort, the prevalence of heart failure and of history of myocardial infarction was very low, respectively 6.3% and 6.1%.

IGFBP7 and the other 2 biomarkers, GDF-15 and P1NP, were chosen since they covered different aspects of cardiac diseases, such as inflammation, apoptosis, fibrosis, and were described as specifically linked to one or more cardiac phenotypes. IGFBP7, a novel prognostic biomarker for heart failure, has been suggested also as a marker for diastolic dysfunction in patients with heart failure with preserved EF, at risk of disease progression [6, 22, 23]. In PREDICTOR, IGFBP7 showed the best association with all cardiac phenotypes. This finding in community-dwelling elder individuals is in agreement with previous studies in patients [13–15].

Some features are worth mentioning. IGFBP7 and GDF-15 markedly increased with age, while this trend for P1NP was weaker, with a borderline statistical significance (*p* = 0.035). Females were significantly less frequent in the highest tertile of IGFBP7 and GDF-15, while the opposite was true for P1NP. This last finding is attributable to the loss of estrogen production due to menopause [24].

Serum creatinine, and consequently eGFR, significantly increased over tertiles of IGFBP7 and of GDF-15, but not of P1NP, which was unrelated to serum creatinine. The presence of diabetes mellitus was strongly associated with higher levels of GDF-15 and to a lesser extent of IGFBP7. In a cohort of 4360 Swedish non-diabetic individuals, GDF-15 was shown to be a strong independent predictor of risk of incident diabetes [25]. While the authors reported that the predictive power of GDF-15 was lost beyond 60 years of age, in PREDICTOR, a cohort with a mean age of 73, higher concentrations of GDF-15 were strongly associated with the presence diabetes mellitus.

The trend for P1NP goes in the opposite direction: the prevalence of diabetes mellitus is significantly higher in the lower tertile of P1NP. Indeed, it has been consistently shown that insulin resistance [26] and overt diabetes mellitus decrease circulating concentrations of markers of bone turnover such as P1NP [27, 28].

Unexpectedly, smokers were significantly more frequent in the lower tertile of concentrations of IGFBP7; however, the statistical significance disappeared in the multivariable analysis: younger age of smokers may well explain this univariate association. On the other side, the markedly higher prevalence of smokers in the highest tertile of GDF-15 has been reported in a Framingham cohort of subjects without overt cardiovascular disease [29]. Abundant evidence exists on association of GDF-15 with impaired endothelial function, arterial stiffness [30], carotid plaques [31], and higher coronary calcium scores [32]. The higher prevalence of history of MI, angina pectoris and atrial fibrillation in particular, goes along the same line of evidence on GDF-15, a cytokine produced in cardiovascular cells under the effect of inflammation and oxidative stress.

In a cohort of 228 patients with HFpEF, IGFBP7 and GDF-15 were found to be related to LV structure, function, and to the burden of comorbidities [33]. The results of PREDICTOR confirm the association of both biomarkers with LV structure and function, in particular with an impaired LV filling. In addition, IGFBP7 and GDF-15 showed for the first time to predict 10-year risk of CV and non-CV major events.

Altogether, the evidence on the three circulating biomarkers assayed in the PREDICTOR community dwelling elderly individuals confirms and extends the findings of previous studies, while it supports a comprehensive assessment of the novel molecule IGFBP7 in relation to cardiac function and clinical outcomes.

The following features of PREDICTOR study strengthen the results presented: identification of subjects through the registry of the National Health Service [15], long-term assessment of outcomes through the same administrative registry, central reading of all echocardiographic exams in a single core laboratory under blind conditions, assay of all anonymized plasma samples for the circulating biomarkers in a central laboratory (Roche Diagnostics GmbH, Penzberg, Germany). A limitation of this study is that the predictive analyses have only been performed for the 1715 patients who could be matched to the Hospital Information System. The remaining 14.3% of the cases were not matched, which may introduce some selection bias. Upon comparing the clinical characteristics of the patients with and without FU available, we found that those with FU available had more often a history of atrial fibrillation and a high AHA/ACC class. In addition, we estimated the statistical power to detect any modest effects on clinical endpoints. For IGFBP7, at an alpha of 0.05, we had at least 80% power for all-cause mortality in the full sample if the true HR was 1.40. For GDF-15, we had at least 80% power for all-cause mortality in the full sample if the true HR was 1.35 with an alpha of 0.05. For all cause hospitalization, for both IGFPB7 and GDF15, with an alpha of 0.05 we had at least 80% power if the true HR was 1.20.

The specificity of a biomarker for a defined cardiac phenotype remains to be elucidated. In fact, there is no such thing as pure/isolated fibrosis without some cardiac myocyte injury and inflammatory activation; on the other hand, myocardial hypertrophy coexists with some interstitial fibrosis. Accordingly, IGFBP7 and GDF-15, two molecules found to be related with cancer and not only with cardiovascular disease states, in the PREDICTOR cohort were found to predict not only all-cause mortality, but also the probability of cancer death after full adjustments (Table 5). This evidence is consistent with other community-dwelling elderly studies [34, 35]

### Conclusions

In conclusion, the peculiar feature of the present study is the comparative evaluation of three circulating biomarkers in about 2000 community-dwelling elderly with a follow-up of over 10 years. The significant association of GDF-15 with structural and functional cardiac alterations confirms previous results from the Framingham population [36]. However, similar findings for IGFBP7 in a community based elderly cohort is novel. In addition, the 10-year follow-up allowed to show the independent predictive power of both IGFBP7 and GDF-15, which was markedly reduced if not cancelled by the presence of NT-proBNP and hs-cTnT in the multivariable models. Both IGFBP7 and GDF15 independently predict mortality and hospitalization for heart failure. This suggests that either these two molecules are directly related to outcomes (quite unlikely) or that their effects on outcomes is mediated, at least in part, by unidentified processes. This is consistent with what already found for NT-proBNP and hs-cTnT [15, 37], both reliable readouts of cardiac injury and dysfunction, but not playing causal roles.

## Supplementary Information

**Additional file 1.** Supplemental material.

## Data Availability

The data are stored at the Department of Epidemiology-Regional Health Service in Rome, at the S. Giovanni-Addolorata Hospital in Rome, and at the Mario Negri Institute for Pharmacological Research in Milan. For privacy policies of the PREDICTOR study, data sharing is not possible.

## Abbreviations

AF: Atrial fibrillation
AHA/ACC: American Heart Association/American College of Cardiologists
BSA: Body surface area
COPD: Chronic obstructive pulmonary disease
CV: Cardiovascular
EDTA: Ethylendiamine tetraacetic acid tripotassium salt
EF: Ejection fraction
eGFR: Estimated glomerular filtration rate
FU: Follow-up
GDF15: Growth differentiation factor-15
HF: Heart failure
HFpEF: Heart failure with preserved ejection fraction
HIS: Hospital information system
HR: Hazard ratio
hs-cTnT: High sensitive cardiac Troponin-T
IGFBP7: Insulin-like growth factor-binding protein-7
LAA: Left atrial area
LV: Left ventricle
LVH: Left ventricular hypertrophy
MFS: Midwall circumference shortening
MI: Myocardial infarct
NT-proBNP: N-terminal brain natriuretic peptide
P1NP: Propeptide of type I procollagen
PICP: Procollagen type I carboxy-terminal propeptide
PIIINP: Amino-terminal propeptide type III procollagen
PREDICTOR: Valutazione della PREvalenza di DIsfunzione Cardiaca asinTOmatica e di scompenso caRdiaco
