# Supplementary material for: IGFBP7 and GDF-15, but not P1NP, are associated with cardiac alterations and 10-year outcome in an elderly community-based study
Source: BMC Cardiovasc Disord. 2021 Jul 3;21:328. doi: 10.1186/s12872-021-02138-8 (PMC8254994; doi:10.1186/s12872-021-02138-8)
Supplement: Supplementary file 1 — Additional file 1. Supplemental material. [file 12872_2021_2138_MOESM1_ESM.docx]

**IGFBP7 and GDF-15, but not P1NP, are associated with cardiac alterations and 10-year outcome in an elderly community-based study**Jennifer M.T.A. Meessen^1^, Giulia Cesaroni^2^, Gian F. Mureddu^3^, Alessandro Boccanelli^4^, Ursula-Henrike Wienhues-Thelen^5^, Peter Kastner^5^, Maria L. Ojeda-Fernandez^1^, Deborah Novelli^1^, Gianfranco Bazzoni^6^, Maurizio Mangiavacchi^7^, Nera Agabiti^2^, Serge Masson^8^, Lidia Staszewsky^1^, Roberto Latini^1^, on behalf of the PREDICTOR Investigators.

**Supplemental Material**

**Methods**

**Echocardiography and cardiac phenotype**

Echocardiograms were recorded using standard DICOM format on digital supports and sent to a central laboratory (European Imaging Laboratory, Rome) for reading. LV mass was calculated according to the recommendations of the American Society of Echocardiography (ASE) and the European Association of Echocardiography (EAE).^^[[1]](#footnote-1)^^ Left ventricular hypertrophy (LVH) was defined as LVmass/body surface area (BSA)>95 g/m2 in women and >115 g/m2 in men. LV mid-wall dysfunction was defined as MFS <15%; this cut-off level is commonly used in studies of high-risk patients^^[[2]](#footnote-2)^^ and has demonstrated prognostic relevance in hypertensive subjects.^^[[3]](#footnote-3)^^ E/e’ parameters and <LA enlargement were measured and considered according the AHA/ACC/ESC guidelines.^^[[4]](#footnote-4)^^ Recommendations for cardiac chamber quantification by echocardiography in adults: an update from the American Society of Echocardiography and the European Association of Cardiovascular Imaging.

Stages of HF were defined according to the criteria of the American Heart Association/American College of Cardiology (AHA/ACC).^^[[5]](#footnote-5)^^ Stage A of HF was defined in the presence of cardiovascular risk factors without evidence of signs or symptoms; stage B was defined as asymptomatic left ventricular dysfunction (ALVD) or demonstrated LV hypertrophy. Stage C indicate patients with current or past symptoms of HF associated with underlying structural heart disease, and Stage D designates patients with truly refractory HF who might be eligible for specialized, advanced treatment strategies, such as mechanical circulatory support, procedures to facilitate fluid removal, inotropic infusions, or cardiac transplantation or innovative or experimental surgical procedures, or for end-of-life care, such as hospice.

1. Lang RM *et al.* Recommendations for chamber quantification. Eur J Echocardiography. 2006; 7:79-108. [↑](#footnote-ref-1)
2. Borlaug BA & Kass DA. Invasive hemodynamic assessment in heart failure. Heart Fail Clin. 2009; 217-28. [↑](#footnote-ref-2)
3. de Simone G *et al*. Influence of obesity on left ventricular midwall mechanics in arterial hypertension. Hypertension. 1996; 276-83. [↑](#footnote-ref-3)
4. Nagueh SF *et al*. Recommendations for the evaluation of left ventricular diastolic function by echocardiography: an update from the American Society of Echocardiography and the European Association of Cardiovascular Imaging. J Am Soc Echocardiogr. 2016; 29 : 277-314. [↑](#footnote-ref-4)
5. Hunt SA *et al.* 2009 Focused update incorporated into the ACC/AHA 2005 Guidelines for the diagnosis and management of heart failure in adults. J. Am. Coll. Cardiol. 2009. [↑](#footnote-ref-5)
